# Modelling aboveground forest biomass using airborne laser scanner data in the miombo woodlands of Tanzania

**DOI:** 10.1186/s13021-015-0037-2

**Published:** 2015-12-02

**Authors:** Ernest William Mauya, Liviu Theodor Ene, Ole Martin Bollandsås, Terje Gobakken, Erik Næsset, Rogers Ernest Malimbwi, Eliakimu Zahabu

**Affiliations:** 1 Department of Ecology and Natural Resource Management, Norwegian University of Life Sciences, P.O. Box 5003, 1432 Ås, Norway; 2 Department of Forest Mensuration and Management, Sokoine University of Agriculture, P.O. Box 3013, Morogoro, Tanzania

**Keywords:** Parametric models, Prediction accuracy, Non-parametric models, LMM, *k*-NN, Sampling design

## Abstract

**Background:**

Airborne laser scanning (ALS) has emerged as one of the most promising remote sensing technologies for estimating aboveground biomass (AGB) in forests. Use of ALS data in area-based forest inventories relies on the development of statistical models that relate AGB and metrics derived from ALS. Such models are firstly calibrated on a sample of corresponding field- and ALS observations, and then used to predict AGB over the entire area covered by ALS data. Several statistical methods, both parametric and non-parametric, have been applied in ALS-based forest inventories, but studies that compare different methods in tropical forests in particular are few in number and less frequent than studies reported in temperate and boreal forests. We compared parametric and non-parametric methods, specifically linear mixed effects model (LMM) and *k*-nearest neighbor (*k*-NN).

**Results:**

The results showed that the prediction accuracy obtained when using LMM was slightly better than when using the *k*-NN approach. Relative root mean square errors from the cross validation was 46.8 % for the LMM and 58.1 % for the *k*-NN. Post-stratification according to vegetation types improved the prediction accuracy of LMM more as compared to post-stratification by using land use types.

**Conclusion:**

Although there were differences in prediction accuracy between the two methods, their accuracies indicated that both of methods have potentials to be used for estimation of AGB using ALS data in the miombo woodlands. Future studies on effects of field plot size and the errors due to allometric models on the prediction accuracy are recommended.

## Background

Estimation of aboveground biomass (AGB) in tropical forests is important for generating information needed for sustainable forest management and understanding the contribution of tropical forests in the global carbon cycle. Particularly in the latter context, estimates of AGB are needed as a primary variable for establishing the increments or decrements in carbon stored in tropical forests, which is typically converted from AGB by using a factor of 50 % or less [1]. In the recent decade, reducing emissions from deforestation and degradation (REDD+), a program under United Nations Framework Conventions on Climate change, has motivated large-scale forest carbon inventories in tropical forests. REDD+ aims to provide positive incentives for developing countries to initiate activities related to reducing carbon emissions, sustainable forest management, and enhancement of forest carbon stock [2]. Unlike other conservation projects, REDD+ is results based, which means that financial benefits rely on forest carbon stock changes that are measured, reported, and verified (MRV). Thus, establishing effective MRV systems that comply with the guidelines of the Intergovernmental Panel on Climate Change, is considered as an integral part of REDD+ implementation [3].

In Tanzania, the National Forestry Resources Monitoring and Assessment (NAFORMA), which is the national forest inventory of Tanzania, has established a total of 30,773 field plots distributed across mainland Tanzania [4]. NAFORMA is expected to be used to produce AGB data for the national forest carbon MRV system necessary for the implementation of REDD+ activities in Tanzania [5, 6]. However, being a field-based inventory, estimates of parameters related to AGB and AGB changes derived from NAFORMA data are not expected to be sufficiently precise to meet the accuracy requirements for a REDD+ MRV system. Therefore, the use of remotely sensed data as auxiliary information is considered as an option towards developing a cost-efficient MRV system in the country.

Airborne laser scanning (ALS) has recently received much scientific and operational attention for estimating AGB than any of the other remote sensing techniques [7]. The potential of ALS has previously been reported in the Nordic countries where it has been used operationally for management inventories for almost 15 years [8]. Recently, promising results from tropical forests [e.g. 9, 10] have also been reported, which have increased the interest in using ALS for REDD+ MRV purposes.

However, large scale AGB assessments with ALS remain challenging due to logistics, cost and the data volume involved if wall-to-wall coverage is to be applied [11]. For such situations, a systematic sampling approach using ALS as a strip sampling tool is a viable option [12]. Within this approach, a collection of ALS measurements are taken along individual flight lines that cover only a small portion of the area of interest. The flight lines are aligned with a network of ground plots [12] which allow the development of statistical models relating the ground reference AGB to metrics derived from coincident ALS data. These models are then used to predict AGB over the entire area covered by ALS strips, and subsequently these predictions are used for final estimation of AGB for the area of interest using either design-based model-assisted or model-dependent inferential frameworks [e.g. 13, 14]. Thus, the quality of the AGB estimates produced by ALS-based inventories relies heavily on the development and application of predictive AGB models.

A review study by Fassnacht et al. [15] shows that the most common prediction methods in ALS-based forest inventories are ordinary least square regression, support vector machines, nearest neighbor-based methods (i.e. *k*-NN and *k*-MSN), and random forest. Of all the methods, ordinary least square regression with stepwise variable selection has been most frequently used for building models between field measurements and ALS metrics [16]. The main advantage of using this type of methodology is the simplicity and clarity of the resulting models [17], especially when the relationship between AGB and the ALS metrics is almost linear. However, fitting and applicability of ordinary least square regression models relies on a number of basic assumptions in relation to the residual distribution which are: independence, normality and constant variance [18]. These assumptions are barely taken into account in most studies [19], especially when dealing with the data that are collected from complex field survey designs that involve clustered observations, repeated measurements, longitudinal measurements, and blocked data. Ignoring the model assumptions when fitting ordinary least square regression models, might lead to spatially correlated errors and consequently, invalid significance tests [20].

Linear mixed effects models (LMMs) offer a modeling and prediction method that is very effective on clustered or spatially correlated data [21, 22]. In addition to accounting for covariates through fixed parameters as in ordinary least square regressions, mixed effects models can also account for various sources of heterogeneity and randomness in the data caused by known and unknown factors by means of random parameters. Application of LMMs are however limited in ALS-based inventories as compared to other prediction methods [23].

Non-parametric approaches, such as *k*-nearest neighbor (*k*-NN) are also considered as an alternative to ordinary least regression, since they do not rely on any distributional assumptions of the data [24]. Thus, *k*-NN is a highly relevant alternative to deal with non-linear and possibly diverse relationships between independent and dependent variables. Furthermore, like other nearest neighbor techniques, *k*-NN allows for both univariate and multivariate predictions of continuous and categorical variables [25, 26]. In forest inventory applications, *k*-NN approaches have been frequently applied in model-dependent frameworks with good results [27] and have also been used for mapping of various forest attributes [28, 29]. Several studies [e.g. 30–32] have compared the performance of *k*-NN with ordinary least square regression (OLS) models in temperate and boreal forests, but few studies have compared LMMs with *k*-NN, especially in the context of the ALS-based inventory. Of particular interest is application and validation of such techniques in the tropical dry forests of Africa, where the application of statistical methods commonly used in ALS-based forest inventories are still limited compared with temperate and boreal forests. Given the growing potential of the use of national forest inventory data and ALS auxiliary information for supporting REDD+ activities in tropical forests [e.g. 6, 33], it is important to explore modeling methods that fully utilize the attributes of design as a fundamental step towards reliable and accurate estimation of AGB using ALS.

Irrespective of the method used, stratification and post-stratification have been considered as effective tools for improving precision of estimates in ALS-based inventories [34]. Stratification according to forest age and/or site quality is commonly used in boreal forests [e.g. 35, 36]. In highly heterogeneous tropical forests, stratification/post-stratification based on vegetation types have been considered as a viable and practical option [37]. However, due to practical limitations, few studies have attempted to assess the effects of stratification and post-stratification on the prediction accuracy and thus on final estimates in tropical forests. For example, in most of the previous studies only a limited number of field plots were available for AGB modeling due to issues such as accessibility and cost. Thus, stratification or post-stratification of the study areas has not been regarded as viable since it could lead to even smaller sample sizes per stratum, making it difficult to fit reliable statistical models for each class [38]. In such situations, most of the previous studies opted to combine sample plot data across classes, for example vegetation types, thus ignoring the effect of vegetation types and associated information.

Our study was conducted in the tropical forests of southern Tanzania which is mainly dominated by the miombo woodlands, along with some forest, cultivated land, and other vegetation types. Miombo woodlands occupy a substantial area of forest land in Tanzania (92 %) [4] and extend to six other countries in sub-Saharan Africa, including Angola, Zimbabwe, Zambia, Malawi, Mozambique, and Democratic Republic of Congo [39]. From a global perspective, miombo woodlands have received considerable attention in the last decade because of its potential to act as a reservoir of belowground and aboveground carbon stocks [40]. Biodiversity is also significant in the miombo woodlands with an estimate of 8500 species of higher plants and more than half of them are endemic [39]. Application of ALS in such areas represents the typical challenge that would be expected when using ALS data for modelling AGB in tropical forests with a high number of species, and diverse vegetation and land use types. The main objective of our study was to assess the performance of parametric and non-parametric methods for modeling and prediction of AGB using ALS data. As a secondary objective, we also assessed the effects of post-stratification by vegetation and land use types on the prediction accuracy of the parametric models.

## Results

### Performance of the parametric and non-parametric methods

**Fig. 1:**
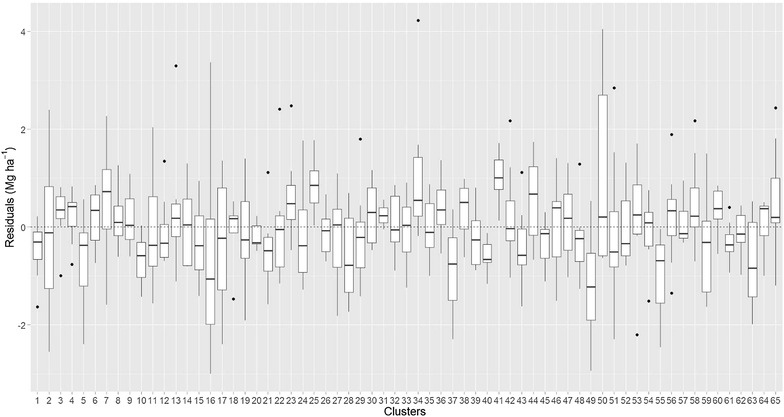
*Box plot* of residuals in the OLS model. The *y axis* shows the value of the residuals and the *horizontal axis* the clusters

The OLS model with square root transformed response variable was selected for building up LMMs. The model contained eight explanatory variables consisting of both height percentiles and canopy density metrics selected using the best subset procedure. The OLS model showed cluster effects on the residual distributions as illustrated in Fig. 1. Some clusters displayed residuals that were above, and some below the zero line, indicating that cluster effects should be accounted for in the modelling. Comparison of the OLS model (Model 1) and the LMM (Model 2) using likelihood ratio test suggested a statistically significance difference (*p* < 0.001) between the two models. Model 2 was considered to have better fit with smaller value of AIC as compared to Model 1.

Re-fitting Model 2 with different correlation structures (i.e., spatial autocorrelation functions and compound symmetry correlation structures) did not significantly improve model fit. The AIC values did not improve when compared to the values in the model without the autocorrelation functions (i.e., Model 2). Furthermore, the likelihood ratio test indicated that there was no statistical significance difference between the models with and without correlation structure (*p* > 0.05). This may also indicate that there is no spatial autocorrelation of the residuals within the clusters. Modelling the residual variance at the cluster level by using variance function (*varIdent*) improved the model performance as measured by the AIC. The likelihood ratio test indicated a statistically significant difference (*p* < 0.0001) between Model 2 and Model 3. The standard errors of the parameters for Model 3 were smaller compared to the other models (Table 1). The quality of Model 3 was further analyzed by comparing an intercept model of Model 3 residuals and a similar model with a random intercept by means of the likelihood ratio test. The test indicated that the two models were not statistically significantly different from each other (*p* > 0.05), implying that Model 3 has successfully accounted for the dependency and heteroscedasticity in the data, therefore the residuals can be considered as independent.

**Table 1:** Parameter estimates and standard errors of the tested models

Covariate^a^	Model 1		Model 2		Model 3	
	Estimate	Standard error	Estimate	Standard error	Estimate	Standard error
Intercept	0.8630	0.2520	0.7385	0.2848	0.6702	0.2238
PF70	0.3481	0.0338	0.3515	0.0345	0.3701	0.0273
TF0	3.7779	0.5713	3.8436	0.6003	4.4363	0.4829
TF5	4.0742	1.2839	3.7574	1.2964	1.7055	1.0384
TF8	−6.2178	1.9462	−6.5277	1.9310	−4.1964	1.4833
PL20	0.3191	0.1241	0.2684	0.1232	0.1844	0.1100
PL30	−0.2295	0.1110	−0.1845	0.1096	−0.1059	0.0951
TL7	−12.4990	4.0241	−11.9814	4.0086	−6.8397	2.7174
TL8	18.3840	5.5361	19.5302	5.4729	10.8928	3.7172

^a^PF70 = Percentile of the first echo canopy heights for 70 % (m); PL20 and PL30 = Percentiles of the last echo canopy heights for 20 and 30 % (m); TF0, TF5, and TF8 = Canopy densities corresponding to the proportion of first echoes above fraction #0 (1.3 m), #5, and #8 (see text); TL7 and TL8 = Canopy densities corresponding to the proportion of last echoes above fraction #7 and #8 (see text)

**Fig. 2:**
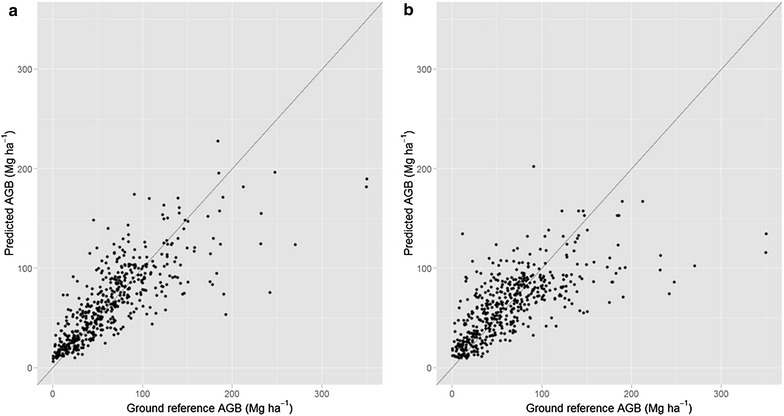
Relationship between ground reference and predicted AGB for LMM (**a**) *k*-NN (k = 10) (**b**)

The *k*-NN imputation tested with different values of *k* ranging from 1 to 10, have shown that, *k* = 10 was the optimal choice with relatively smaller RMSE_CV_ % value. We further tested the dependency and heteroscedasticity of the residuals obtained from best *k*-NN imputation (i.e., k = 10), by comparing two residual models using the likelihood ratio test (i.e., a residual intercept model and a random intercept model). The results from the likelihood ratio test showed that there was no statistically significant differences between the two models. Comparing the results of the best parametric model (i.e., Model 3) and the non-parametric (i.e., *k* = 10) (Table 2), our results suggest that the parametric models performed well in our dataset as indicated by both R^2^ and RMSE_CV_ %. Graphical illustrations for the performances of the two methods are presented in Fig. 2.

**Table 2:** Predictors, pseudo R-squared (R^2^), and relative root mean square error from the cross validation (RMSE_CV_ %) of the two prediction methods

Prediction method	Predictors^a^	R^2^	RMSE_CV_ %
LMM (Model 3)	PF70, TF0, TF5, TF8, PL20, PL30, TL7, TL8	0.69	46.8
*k*-NN (k = 10)	PL80, TF0, TL2, PF80, TL4, TL8, PS40, TL5, TF2, TL7, TL6, PF70, MaxF, PL90, PL50, MeanL, TF7, PF10, PL60, TF3	0.58	55.9

^a^PF10, PF70, and PF80 = Percentiles of the first echo canopy heights for 10 %, 70 %, and 80 (m); PL20, PL30, PL40, PL50, PL60, and PL90 = Percentiles of the last echo canopy heights for 20, 30, 40, 50, 60, and 90 % (m); TF0, TF2, TF3, TF5, TF7, and TF8 = Canopy densities corresponding to the proportion of first echoes above fraction #0 (1.3 m), #2, #3, #5, #7, and #8;TL2, TL4, TL6, TL7, and TL8 = Canopy densities corresponding to the proportion of last echoes above fraction #2, #4, #6, #7, and #8; MaxF and MeanL = Maximum and Mean of the canopy height distributions of the first and last echoes, respectively

### Effect of post-stratification on prediction accuracy of the parametric models

To account for the effect of post-stratification on model accuracy, we assessed the performance of the parametric model (i.e., Model 3) on different vegetation and land use types (Table 3). The results indicated that there were variations in the prediction accuracy of the model across the categories. The RMSE % and RMSE_CV_ % of Model 3 (i.e., non-post-stratified model) varied from one category to another; smaller values of RMSE % and RMSE_CV_ % were reported for vegetation types as compared to land use types.

**Table 3:** Performance of the parametric model (Model 3) on different vegetation and land use types

Category^a^	RMSE (Mg ha^−1^)	RMSE_CV_ (Mg ha^−1^)	RMSE %	RMSE_CV_ %
Vegetation type				
Forest	22.7	25.1	25.7	28.4
Woodlands	29.4	31.6	44.3	47.7
Other cover types	37.6	38.5	80.2	82.1
Land uses type				
Production and protection forests	30.4	31.1	40.2	41.1
Wildlife reserves	25.5	26.3	50.2	52.0
Agriculture and other land use types	33.2	34.0	73.0	74.7

^a^Forest = land spanning more than 0.5 ha with trees that have heights of more than 5 m and a canopy cover of more than 10 %. It does not include land that is predominantly under agricultural or urban land use. Woodland = forestland with less dense canopy cover compared to forest. Other cover types = all cover types that were neither forest nor woodlands. Production and protection forests = forest areas designated for protection of water (i.e. catchment forests) and that designated for production of wood, respectively. Wildlife reserves = forest areas designated for game reserves and game controlled areas. Agriculture and other land use types = areas designated primarily for a function other than production, protection or game reserves. Details descriptions of these categories are given in MNRT [60]

**Fig. 3:**
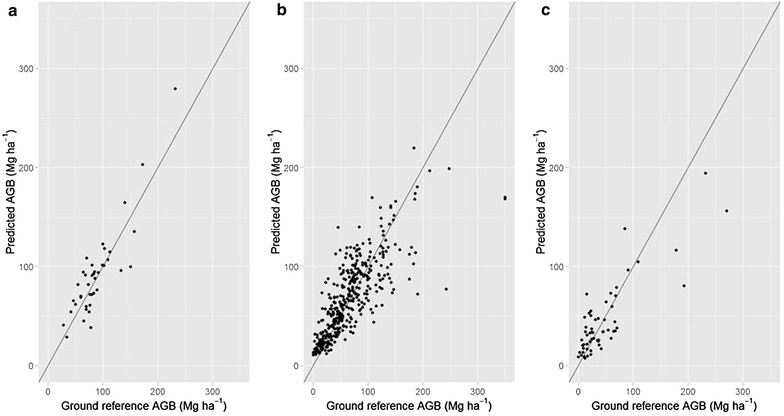
Relationship between ground reference and predicted AGB for LMM in forest (**a**) woodlands (**b**) other cover types (**c**)

**Fig. 4:**
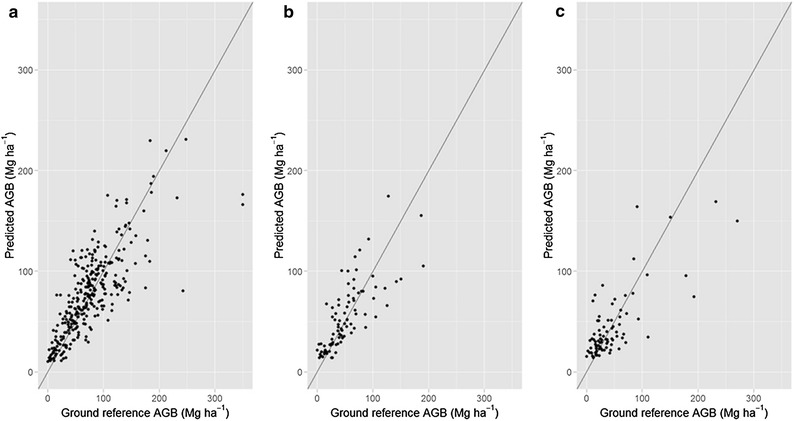
Relationship between ground reference and predicted AGB for LMM in production and protection forests (**a**) wildlife reserves (**b**) agriculture and other land uses (**c**)

Separate random intercept models were fitted for each of the categories (Table 4) and compared with the non-post-stratified model presented in the previous section. Generally, the RMSE_CV_ % for the post-strata models were relatively small compared to the values obtained when evaluating the non-post-stratified model across respective post-strata. The accuracy of the post-strata models varied in terms of model fits (i.e., R^2^) and RMSE_CV_ % depending on the vegetation and land use types (Table 4). Graphical plots in Figs. 3 and 4 illustrate the performance of the post-strata models.

**Table 4:** Predictors, number of observations (n), pseudo R-squared (R^2^), and relative root mean square error from the LOOCV (RMSE_CV_ %) for separate LMM fitted for different vegetation types and land use types

Category^a^	Predictors^b^	n	R^2^	RMSE_cv_ %
Vegetation type				
Forest	PF20, MaxL, MeanL, PL10, PL40, PL70, PL90, TL9	40	0.85	23.9
Woodlands	PF70, TF0, PL20	391	0.63	45.3
Other cover types	MeanF, PF20, PF60, TF5, MaxL, PL70	58	0.83	68.3
Land use type				
Production and protection forests	PF40, PF60, PF70, TF0, TF9, PL20	314	0.64	40.8
Wildlife reserves	CVF, PF20, PF90, TF5	91	0.73	49.8
Agriculture and other land uses	TF1, TF4, MaxL, MeanL, CVL, TL0, TL4	84	0.69	68.0

^a^Forest = land spanning more than 0.5 ha with trees that have heights of more than 5 m and a canopy cover of more than 10 %. It does not include land that is predominantly under agricultural or urban land use. Woodland = forestland with less dense canopy cover compared to forest. Other cover types = all cover types that were neither forest nor woodlands. Production and protection forests = forest areas designated for protection of water (i.e. catchment forests) and that designated for production of wood, respectively. Wildlife reserves = forest areas designated for game reserves and game controlled areas. Agriculture and other land use types = areas designated primarily for a function other than production, protection or game reserves. Details descriptions of these categories are given in MNRT [60]

^b^PF20, PF40, PF70, and PF90 = Percentiles of the first echo canopy heights for 20 %, 40 %, 70 %, and 90 (m); PL10, PL20, PL70, and PL90 = Percentiles of the last echo canopy heights for 10, 20, 70, and 90 % (m); TF0, TF1, TF4, and TF5 = Canopy densities corresponding to the proportion of first echoes above fraction #0 (1.3 m), #1, #4, and #5; TL0, TL4, TL6, TL7, and TL8 = Canopy densities corresponding to the proportion of last echoes above fraction #0 (1.3 m), #4, #6, #7, and #8; MeanF and MeanL = arithmetic mean of first or last echo laser canopy heights, respectively (m); MaxF and MaxL = maximum of first or last echo laser canopy heights, respectively (m); CVF and CVL = Coefficient of variations for the first and last echo laser canopy heights, respectively

## Discussion

Our study aimed at comparing the performance of the parametric (LMMs) and non-parametric (*k*-NN) methods for predicting AGB using ALS data in the miombo woodlands of Tanzania. Effects of post-stratification by vegetation and land use types on the prediction accuracy of the parametric method were considered as the secondary objective. The findings from this study demonstrate that both LMMs and *k*-NN are suitable methods for predicting AGB using ALS data. To our understanding this is one among the early studies attempting to use ALS in the miombo woodlands of Tanzania. Thus the findings of this study open up methodological insights on the use of ALS as tool for AGB assessment in similar type of vegetation in sub-Saharan Africa.

Specifically, the findings have shown that LMM is the best prediction method; by allowing the specific field sampling design to be accounted for in the modeling, but also by having slightly higher prediction accuracy compared to *k*-NN. This is not surprising, and has been reported in most of the studies that have attempted to compare parametric and non-parametric methods in prediction of various forest attributes [e.g. 30, 41, 42]. However, the strength of *k*-NN is that it was able to account for the dependence and heteroscedasticity in the data. This indicates that it can reliably be used for estimation and making inference when deemed necessary, especially in the design-based framework of forest inventory [e.g. 43, 44] with non-parametric based estimators (e.g. difference estimator).

Results based on the LMM illustrate that incorporating the cluster structure by using variance function in the model selection process can result in a model with better fit, as supported by the likelihood ratio test. This implies that it is the between-cluster variability that should be considered when calibrating the ALS models using NAFORMA data, rather than the within-cluster variability. By modelling the residual variance per cluster through an appropriate variance structure (i.e., the *varIdent* structure) we were able to account for this variability in the data, which resulted in a model with smaller standard errors for the parameter estimates as compared to the other tested models (Table 1). Smaller standard errors of the parameter estimates (Table 1) indicate that the model is more efficient when predicting outside the sample. Furthermore, smaller standard errors of the parameter estimate is an important property in improving precision of the estimates, especially when making inference using model-based estimators [45] which theoretically rely on the quality of the model parameters.

Further evaluation of the best non-post-stratified model across post-strata, showed that there were variations in prediction accuracy across different vegetation and land use types. This could likely be attributed to the difference in stem diameter and the number of trees per unit area, which entirely affect the distribution of AGB and the characteristics of the ALS data in each of the post-strata. When fitting separate models by post-strata, our findings showed that that there was a slight gain in prediction accuracy compared to the use of non-post-stratified model in the respective post-strata. This might be due to the homogeneity of the respective category, which in turns improves the relationship between ALS metrics and the ground reference AGB. For example, in the post-strata such as forest, or production and protection forests where the distribution of AGB is characterized by trees with high canopy cover and more uniform stems, the RMSE_CV_ % were relatively smaller compared to other categories. On the other hand, the higher RMSE_CV_ % value in the agricultural and other land uses might be attributed to the fact that most of the trees in this category are scattered with sparse canopy, and the tree crowns are smaller with some appearing to be in a degraded form.

Although post-strata models performed well compared to non-post-stratified models, their practical application in the miombo woodlands poses a number of challenges when used for estimation and inference. Based on the sampling design described in this study, the use of post-strata models would require having thematic maps for the land use classes and vegetation types. Such maps are not trivial to produce, and our results (not presented) indicated that the classification accuracies vary substantially among these categories. Thus, since the difference between the non-post-stratified model and the post-strata models were modest, we would rather recommend the non-post-stratified model (which disregards the land use and vegetation types) to be more adequate for most applications that will involve large-scale AGB estimation supported by ALS data, at least until high quality thematic maps are made available.

Generally, the finding of our study in terms of model quality criteria such as R^2^ and RMSE_CV_ % for non-post-stratified and post-strata models are in line with most of the published studies from tropical forests [46–49]. The majority of these studies reported R^2^ ranging from 64 % [50] to 90 % [51]. Similarly, a study by Asner et al. [47] across four tropical regions in Panama, Peru, Madagascar, and Hawaii reported R^2^ ranging from 0.68 to 0.85. Recently, a study from the tropical rainforest of Tanzania by Hansen et al. [9] reported RMSE_CV_ % ranging from 32.3 to 36.8 % for models with different forms and different sets of predictor variables. However, direct comparison with these results should be taken with caution due to the wide range of variations existing in the tropical forests, along with the different sample sizes and plot sizes used in different studies.

Even though we are convinced that our findings reflect the potential performance of ALS-based AGB models in dry tropical forest conditions, but there might be ways to further improve the quality of the models. For instance, the plot size used in our study was relatively small compared to what has been used in the studies that reported higher prediction accuracy [e.g. 47, 52]. Most of these studies used plot sizes that are even twice as large as used in the current study. For example, Mauya et al. [53] reported a decrease in RMSE_CV_ % from 63.6 to 29.2 % for plot sizes ranging from 200 to 3000 m^2^ in a high-biomass rainforest. The increase in prediction accuracy for studies based on larger plots might be attributed to the so-called spatial averaging of the errors, because both the field observations and the ALS data capture more of the spatial variation and are closer to the average value [e.g. 9, 54]. Furthermore, the relative and negative influence of plot positioning error on the prediction accuracy is reduced for the larger plot sizes, because the overlap between the field and ALS-data becomes larger as plot size increases [55]. In addition, plot boundary effect which has potential to cause discrepancies between field and ALS-based measurements, is reported to be relatively smaller for the larger plots compared to the smaller plots [53].

The concentric design of the field plots used in the current study also introduced errors in the relationships between AGB estimated on the plots and the ALS data. With this design, small trees are measured only in the center of a field plot while the largest trees are measured across the entire plot. However, smaller trees are also found in the outer part of a plot, and these trees will be measured by the laser but not recorded in the field data. Measuring all trees across a plot would clearly improve model fit. However, this study focused on the already existing design and data established by NAFORMA, thus it was also important to demonstrate how the NAFORMA data would be used with ALS auxiliary information. Similarly, miombo woodlands are dominated by a lower herbaceous layer of the vegetation which was not accounted for in the field measurements but were captured by the ALS data. Although a threshold of 1.3 m was applied to the ALS data to define the “canopy” layer, it is likely that the ALS data contains height observations reflected from vegetation that are not recorded by the tree measurements. This has certainly introduced additional errors into the models and reduced their performance. Lastly, it should be mentioned that the errors associated with the allometric models used to compute AGB (which were ignored in this study) will tend to affect the accuracy of the ALS-based prediction models. A general model by Mugasha et al. [56] that combines all the tree species was used to compute AGB on the ground plots. Given the high number of tree species it is likely that the uncertainty of the field-based reference values is substantial. To what extent this error affects the prediction accuracy of the ALS-based models is still unknown in the miombo woodlands and should be the focus of future research.

## Conclusion

To conclude, our study demonstrated that predicting AGB using ALS data can be reliably done in the miombo woodlands of Tanzania. Our results on the comparison of the prediction methods have shown that LMM is the most appropriate method for AGB prediction using ALS data, as indicated by RMSE_CV_ %, but also by considering its strength of accounting for the complex sampling design of the NAFORMA program. The prediction accuracy of *k*-NN was relatively smaller compared to LMM, yet it can be used when there is a need for using non-parametric method. Post-stratification by vegetation types seemed to favor the prediction accuracy compared to land use types. However, the non-post-stratified model has relatively more advantages due to its versatility and practical limitations of using post-strata models. Thus, we suggest using LMM (i.e., Model 3) that combines all the post-strata for applications involving large-scale AGB estimation in the future. Lastly, our study identified important knowledge gaps and directions of future research, such as assessing the effects of field plot size and the use of on-plot protocols which is based on complete census of all the trees in a plot, rather than a sample according to tree size. Finally, a better understanding and quantification of the effects of allometric model errors on overall uncertainties of ALS-based models and AGB estimates is a fundamental topic for future studies.

## Methods

### Study area

**Fig. 5:**
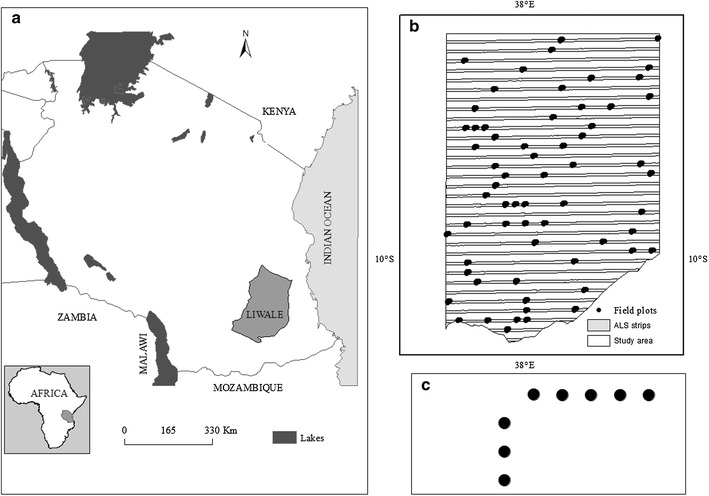
Map showing location of the study area in Liwale, southern Tanzania (**a**), distribution of clusters of field plots and ALS strips in the study area (**b**) and the cluster structure with distribution of individual field plots within a cluster (**c**)

The study area is located in Liwale district (9°54′S, 37°38′E) (Fig. 5a), Lindi region, Tanzania, and has a total size of 15,867 km^2^ (Fig. 5b). The mean annual temperature of Liwale district ranges between 20 and 30 °C. Rainfall pattern is bi-modal with a dry season from June to October. A short rainy period usually starts in late November and lasts until January. There is dry spell in February followed by a longer wet season which lasts from March until May. The mean annual rainfall ranges from 600 to 1000 mm [57]. The study area contains typical miombo flora of high trees with shrubs and grasses on the forest floor. In general, the area is characterized by a high species diversity associated with typical miombo tree species such as *Brachystegia* sp., *Julbernadia* sp., and *Pterocarpus angolensis*.

### Sampling design

The field plots used in this study were initially established by NAFORMA in 2011. The sampling design used by NAFORMA is double-sampling for stratification which was designed based on a simulation study described by Tomppo et al. [6]. The first-phase sample consists of clusters of plots on a 5 × 5 km grid over mainland Tanzania. The first-phase clusters were stratified based on predicted growing stock, time consumption for cluster measurements and slope of the terrain [6]. All together, the first-phase clusters that contain 6–10 plots (Fig. 5c) per cluster were assigned to 18 pre-defined strata. The second-phase samples were systematically selected from the first phase sample, with different sampling intensities in each of the 18 strata following an optimal allocation procedure [58] and with cost functions tailored for each stratum. Greater sampling intensity was allocated to strata with large predicted growing stock, and smaller sampling intensity to strata with small predicted growing stock. Only the clusters selected during the second phase of sampling were measured in the field. The distance between field plots within a cluster was 250 m, while the distance between clusters varied from the shortest possible distance (5 km) to 45 km.

### Field measurements

NAFORMA field plots were revisited during the first quarter of 2012. The aim of the field work was to accurately record the positions of the field plots and update the field information to have temporal consistence between field measurements and the time of acquiring ALS data. Measurements on the plots followed the same procedure used by NAFORMA in 2011. The circular plots of 15 m radius were identified. Diameter at breast height (*dbh*) was measured using caliper or diameter tape, following the lower *dbh* thresholds in accordance with the concentric circle plot design. The radii of the concentric circles were 2, 5, 10, and 15 m, respectively. Trees with *dbh* ≥ 1, ≥ 5, ≥ 10, and ≥ 20 cm, respectively, were measured and assigned to these concentric plots.

Species names were recorded for every tree measured for *dbh*. Every fifth tree in the cluster was selected as a sample tree and measured for height using Suunto hypsometer. The heights of the remaining trees were predicted using diameter-height models that were developed based on the sample trees. Differential Global Navigation Satellite Systems were used to calculate the coordinates of the center point of each sample plot. Two Topcon Legacy 40-channels dual frequency receivers observing both pseudo-range and carrier phase of the Global Positioning System (GPS), along with the Global Navigation Satellite System (GLONASS) were used as rover (on the plot) and base station, respectively. Based on the positional standard errors reported by Pinnacle [59], the estimated accuracy of the planimetric plot coordinates ranged from 0.004 to 1.334 m, with an average of 0.194 m.

Tree AGB was estimated using allometric models for miombo woodlands developed by Mugasha et al. [56]. The AGB estimates of the individual trees were then summed for each plot, and scaled to per-hectare values according to their respective plot area determined by *dbh*-thresholds. The plots were grouped according to their respective stratum, land use, and vegetation types following the procedure described by NAFORMA in MNRT [60]. In this study, we narrowed the land use classes and the vegetation types described in MNRT [60] into three categories to simplify the interpretation of the results, but also to have enough samples for each category (Table 5). The land use classes were grouped into: (1) production and protection forests; (2) wildlife reserves; (3) agricultural and other land use types. Similarly, the vegetation types were grouped into: (1) forest; (2) woodlands; (3) other cover types.

**Table 5:** Summary of field data

Category^a^	Number of plots	AGB (Mg ha^−1^)			
		Minimum	Maximum	Mean	Standard deviation
Stratification					
Stratum 5	116	3.9	158.2	45.9	29.8
Stratum 6	81	2.2	179.2	51.3	41.7
Stratum 7	90	2.4	270.3	84.7	61.3
Stratum 8	119	0.3	349.9	79.4	53.7
Stratum 9	23	0.3	125.6	38.7	35.4
Stratum 10	6	0.3	56.1	16.0	20.5
Stratum 11	16	55.1	186.9	87.9	32.4
Stratum 12	38	34.3	182.7	85.2	35.6
Post-stratification					
Vegetation type					
Forest	40	27.8	232.2	88.3	39.6
Woodlands	391	0.3	349.9	66.3	48.4
Other cover types	58	0.3	270.3	46.9	53.7
Land use type					
Production and protection forests	314	0.3	349.9	75.6	49.5
Wildlife reserves	91	0.3	190.6	50.8	39.0
Agriculture and other land uses	84		270.3	45.5	48.1
All	489	0.3	349.9	65.8	49.2

Number of plots for the different strata and post-strata together with minimum, maximum, and mean ground reference AGB values with their corresponding standard deviation

^a^Stratum 1–6 refers to the strata to which field plots belongs as described in the text and elaborated by Tomppo et al. [6]; Forest = land spanning more than 0.5 ha with trees that have heights of more than 5 m and a canopy cover of more than 10 %. It does not include land that is predominantly under agricultural or urban land use. Woodland = forestland with less dense canopy cover compared to forest. Other cover types = all cover types that were neither forest nor woodlands. Production and protection forests = forest areas designated for protection of water (i.e. catchment forests) and that designated for production of wood, respectively. Wildlife reserves = forest areas designated for game reserves and game controlled areas. Agriculture and other land use types = areas designated primarily for a function other than production, protection or game reserves. Details descriptions of these categories are given in MNRT [60]

### ALS data

Acquisition of the ALS data was carried out along 32 parallel strips with an average width of 1374 m, which were systematically distributed over the study area in an east–west direction. The ALS strips were spaced 5 km apart, following the NAFORMA 5 × 5 km grid. A Leica ALS 70 airborne laser sensor (Leica Geosystems AG, Switzerland), carried by a Cessna 404 aircraft, was used to acquire the data from 10 February to 7 March 2012. The measurements were acquired from an average flying altitude of approximately 1320 m above ground, at an average ground speed of 77.2 ms^−1^. The scanning rate was 36.5 Hz and the instrument operated at a pulse repetition frequency of 193 kHz. The average point density was around 1.8 points m^−2^.

Processing of the ALS data started by classifying the ALS echoes into ground or vegetation echoes using the progressive irregular triangular network densification method [61, 62] implemented in the TerraScan software [62]. A triangular irregular network (TIN) was created using the ALS echoes classified as ground echoes. The heights above the ground surface were then calculated for all vegetation echoes by subtracting the TIN height at their respective xy-positions. Up to five echoes were registered per pulse, but we used only the three echo categories “single”, “first of many”, and “last of many”. The “single” and “first of many” echoes were pooled into one dataset denoted as “first” echoes, and correspondingly, the “single” and “last of many” echoes were pooled into a dataset denoted as “last” echoes.

For each echo category, height distributions were first created as described by Næsset [63]. A height threshold of 1.3 m was applied in order to separate trees from falsely classified ground features and low vegetation. Subsequently, heights at ten percentiles (0th, 10th,…,90th) of these height distributions were computed to represent canopy height distribution and labeled PF0, PF10,…, PF90 (first echoes) and PL0, PL10,…, PL90 (last echoes), respectively. Furthermore, measures of the canopy density were also computed for first and last echoes. The range between the lowest ALS canopy height (>1.3 m) and the 90th percentile height was divided into 10 layers of equal height. Canopy densities were then computed as the proportion of echoes above each layer to total number of first echoes and labeled TF0 (>1.3 m), TF1,…, TF9. Density variables for the last echo distributions were calculated in the same way and labeled TL0, TL1,…,TL9. Furthermore, mean (MeanF and MeanL), maximum (MaxF and MaxL) and coefficient of variation (CVF and CVL) of the canopy height distributions were also computed for both first and last echoes.

### Statistical analyses

#### An overview

Three statistical techniques were used to develop relationships between the ground reference AGB and the ALS metrics. These included OLS, LMMs, and *k*-NN technique.

1. Candidate explanatory variables from the ALS metrics were selected and three OLS model forms relating ground reference AGB and ALS metrics were fitted and tested.
2. The best selected model form from step 1 was used to build LMM with random effect at the cluster level.
3. To account for spatial dependence within the clusters we introduced LMMs with different correlation structures and compared with the LMM fitted in step 2.
4. LMM with variance structure at the cluster level was also fitted. The model was compared with the LMM fitted in step 2 using likelihood ratio testing. The best selected model (i.e., from step 1 to 4) was further evaluated using a cross validation procedure.
5. Finally, the *k*-NN imputations were fitted and compared with the best model selected from the procedure described above using measures of reliability based on cross validation.

### Parametric methods

#### Model development (OLS)

OLS are among the most common methods for modeling and predicting AGB in ALS-based forest inventory. As part of the model development procedure, we first applied an automated approach to select candidate predictor variables using the “*regsubset*” function from the leaps package [64] in the R statistical software [65]. The “*regsubset*” regression performs “all subsets” where all possible variable combinations are considered and ranked based on different scoring criteria (adjusted R^2^, Mallow’s *C*_*p*_ statistics, BIC, etc.) [66]. In this study we used Mallow’s *C*_*p*_ statistics [67], a combination of predictors that minimizes the Mallow *C*_*p*_ over all possible subsets, was considered as the best subset for model development. The variable selection was repeated for log-transformed variables and square root transformed response variable. Thus, three types of OLS models were finally fitted and tested. Of all the three model forms, square root transformation (Eq. 1) was selected as the best based on our initial test results (not presented), i.e.,

1 $$\begin{aligned} \sqrt {y_{j} } &= \beta_{0} + \beta_{1} x_{j1} + \cdots + \beta_{k} x_{jk} + \epsilon_{j} \nonumber \\ j &= 1 \ldots n\quad \epsilon_{j} \sim N \left( {0,\;\sigma_{\epsilon }^{2} } \right) \end{aligned}$$

where *y*_*j*_ is the ground reference AGB of the *j*th sample plot, *x*_*j*1_…,*x*_*jk*_ are the *k* predictor variables (i.e. ALS metrics), *β*_0_,…,*β*_*k*_ are the parameter estimates, *n* is the number of sample plots and *ɛ*_*j*_ is the plot level residuals.

### Model development (LMM)

The sampling design employed by NAFORMA imposes a hierarchical data structure by which the field plots are nested within the clusters. In such a case, LMM is considered as an ideal tool for development of predictive models [22, 68] that accounts for spatial dependence of the plots within the clusters. LMM consists of two main parts; fixed and random effects. The fixed effects are common to all subjects, while random effect parameters are specific to each subject [69]. The predictor variables of the OLS model (Eq. 1) were used as the fixed effects and the cluster number, or identity was used as the grouping variable (random effect), which can also be regarded as subject. The standard form of LMM as applied in this study is:

2 $$\begin{aligned} \sqrt {y_{ij} } &= \beta_{0} + \beta_{1} x_{ij} + \cdots + \beta_{k} x_{ijk} + b_{i} + \epsilon_{ij} \nonumber\\ i &= 1, \ldots ,M\quad j = 1, \ldots ,n_{i} \nonumber \\ \;b_{i} &\sim N\left( {0,\sigma_{b}^{2} } \right)\;\epsilon_{ij} \sim N(0,\sigma_{\epsilon }^{2} ) \end{aligned}$$

where *y*_*ij*_ is the ground reference AGB of $j{\text{th}}$ sample plot in the *i*th cluster, $x_{ij1} , \ldots ,x_{ijk}$ are *k* fixed effects, *β*_0_, … , *β*_*k*_ are the fixed effects parameters, $n_{i}$ is the number of sample plots within the cluster j and *M* is the number of clusters. We assumed that cluster level random effects *b*_*i*_ were independent of the plot level residuals *ɛ*_*ij*_.

To evaluate the significance of the random effect we re-fitted the OLS model using generalized least square *function*, in order to compare the OLS with the LMM using the likelihood ratio tests, as described by Zuur et al. [68].

To account for the non-constant variance and spatial autocorrelation that might not have been accounted for by the random effect, we further refitted the LMM with variance and correlation structures and compared with the LMM (i.e., the random intercept model). The details for this procedure are described below and elaborated further by Zuur et al. [68].

### LMMs with correlation structures

We fitted five different LMMs using maximum likelihood estimation (ML), each assuming different spatial autocorrelation structures (i.e., linear, ratio, exponential, spherical, and Gaussian). This was aimed at testing the effect of spatial autocorrelation to account for field plot proximity within the clusters. In addition, we also tested compound symmetry correlation structure, assuming that correlation among plots within a cluster is constant but might vary from one cluster to another. The LMMs that incorporate spatial autocorrelation and compound symmetry correlation structures were compared with LMM without correlation structure (i.e., the random intercept model) using a likelihood ratio test. Details of these correlation structures are fully described in Pinheiro, Bates [69].

### LMM with variance structure

To account for variation (i.e., heteroscedasticity due to cluster) not accounted for by the random effects, we also re-fitted the LMM (i.e., the random intercept model) assuming that the residuals were independent on cluster level. In this case, we used the *varIdent* variance function implemented in the *nlme* package [69]. The model was fitted using ML, and compared with LMM (i.e., the random intercept model) using the likelihood ratio test to determine the effect of cluster information on the model accuracy. Finally, the best model as indicated by the likelihood ratio test was refitted using restricted maximum likelihood (REML). To ensure that our modelling strategy has accounted for heteroscedasticity due to cluster structure, the residuals from the best model were further analyzed by fitting a residual intercept model (i.e., null model) and a residual random intercept model. The two models were compared using a likelihood ratio test to determine if we still have an effect of cluster structure in the residuals. Pseudo R-square (R^2^) computed as the square of the Pearson correlation coefficient between observed and predicted values was used to assess the quality of the model fit.

### Accuracy assessment

To enable a fair comparison of the best LMM and non-parametric imputations (presented below), the prediction error of the best LMM was estimated by using leave-one-cluster-out cross validation (LOCOCV) [70]. Owing to the number of clusters used in the current study and the lack of an independent validation dataset, LOCOCV was therefore applied. The predicted values of AGB obtained from the LOCOCV $( {\text{i}}.{\text{e}}., \;S\widehat{QR}T(AGB) )$ were corrected for bias (caused by the square root transformation) using the method by Gregoire et al. [71] according to

3 $$\widehat{AGB}_{corrected} = (S\widehat{QR}T(AGB))^{2} + MSE$$

where *MSE* is the mean square error of the model. Relative root mean square error from the LOCOCV (*RMSE*_*CV*_ %) was used as a criterion for assessing model accuracy and calculated as

4 $$RMSE_{CV }\;\% = \frac{{\sqrt {\mathop \sum \nolimits_{i = 1}^{n} \left( {y_{i} - \widehat{y}_{i} } \right)^{2} /n} }}{{\overline{y} }} \times 100$$

where *y*_*i*_ and $\widehat{y}_{i}$ denote ground reference AGB and predicted AGB for plot *i*, respectively, and ${\bar{\text{y}}}$ denotes mean ground reference AGB for all plots. RMSE_CV_ % is a good measure of how accurately the model predicts the response and is the most important criterion for fit if the main purpose of the model is prediction [72].

### Non-parametric method

#### *k* -NN imputation

Imputation using *k*-NN is a non-parametric method that has often been used for predicting various attributes in forest inventories supported by remotely sensed auxiliary information [e.g. 73, 74]. In *k*-NN terminology it is typically distinguished between *reference* and *target* datasets. The population units for which observations of both response and explanatory variables are available is labeled as the reference set; the set of the population units for which only the explanatory variables are available is termed as the target set. In our study, the reference set contained both ground reference AGB and the ALS metrics, while the target set contained only the ALS metrics.

The similarity between the *i*th target observation and *j*th reference observation was quantified by means of the Euclidian distances calculated in the feature space as:

5 $$d_{ij} = \sqrt {(x_{i} - x_{j} )^{'} (x_{i} - x_{j} )}$$

where *x*_*i*_ and *x*_*j*_ are the feature vectors. Hence, the similarity between the target and reference observations will increase as the *d*_*ij*_ distances decrease, and consequently the nearest neighbor of the $i{\text{th}}$ target observation is the reference observation located at the shortest Euclidian distance in the feature space.

The imputed value $\widehat{y}_{i}$ is expressed as a weighted sum of the responses taken from the nearest *k* reference observations as follows:

6 $$\widehat{y}_{i} = \mathop \sum \limits_{j = 1}^{k} w_{ij}\;y_{j}^{i}$$

where *y*_*ij*_^*i*^, *j* = 1, 2,…*k* is the set of the response variable observations for the *k* reference set elements that are nearest to the ith target set elements in the feature space. The *k*-weights associated with the response in Eq. 6 were obtained as

7 $$w_{ij} = d_{ij} \left[ {\mathop \sum \limits_{j = 1}^{k} d_{ij} } \right]^{ - 1}$$

In order to reduce the data redundancy and improve the overall interpretability, a variable selection procedure was applied by using *varSelection* function in *yaImpute* package [75] of the R software [65]. Model fitting was done by using *knnreg* function in *caret* package [76]. For *k*-NN imputations, selection of *k* has an influence on the accuracy of the imputation. Large values of *k* are not recommended since this will shift the predictions towards the sample mean. For this study we tested the values of *k* ranging from 1 to 10 and selected the value with lowest ${\text{RMSE}}_{\text{CV}}\;{\text{\% }}$ obtained from the cross validation. Specifically, we used LOCOCV, where one cluster at time was used as the target set while the remaining clusters were used at the reference set. To assess the ability of the *k*-NN to account for the dependence and heteroscedasticity due to cluster structure, we computed the residuals from the LOCOCV, then we fitted a residual intercept model and compared with residual random intercept model using likelihood ratio test. Lastly, we compared *k*-NN and LMM using RMSE_CV_ %.

### Assessing the effect of post-stratification on prediction accuracy

To account for the variation in prediction accuracy that might be attributed to the differences in vegetation and land use types, the best LMM (i.e., LMM with variance structure) was further evaluated for different vegetation and land use types. Both relative root mean square errors from model predictions (RMSE %) and LOCOCV (RMSE_CV_ %) were calculated and presented for each category of vegetation and land use type. Specific LMMs (i.e., random intercept models) were fitted for the post-strata as defined by vegetation and land use types. The models were evaluated using LOCOCV. For each of the post-stratum model, R^2^ and RMSE_CV_ % were computed and compared with the RMSE_CV_ % obtained when evaluating the non-post-stratified model for the respective post-stratum.

## Abbreviations

ABA: area-based approach
AGB: aboveground biomass
AIC: akaike information criterion
ALS: airborne laser scanning
GLONASS: global navigation satellite system
GPS: global positioning system
*k*-NN: *k*-nearest neighbors
LMM: linear mixed effects model
LMMs: linear mixed effects models
LOCOCV: leave-one-cluster-out cross validation
MRV: measuring, reporting and verification
MSE: mean square error
OLS: ordinary least square
REDD+: reducing emissions from deforestation and forest degradation
RMSE: root mean square error
RMSE_CV_: root mean square error from cross validation
SAR: synthetic aperture radar
SE: standard error
SSE: sum of square error
TIN: triangular irregular network
UNFCCC: United Nations Framework Convention on Climate Change
